# Feasibility and acceptability of PrE-operative Physical Activity to improve patient outcomes After major cancer surgery: study protocol for a pilot randomised controlled trial (PEPA Trial)

**DOI:** 10.1186/s13063-018-2481-2

**Published:** 2018-02-17

**Authors:** Daniel Steffens, Jane Young, Paula R. Beckenkamp, James Ratcliffe, Freya Rubie, Nabila Ansari, Neil Pillinger, Michael Solomon

**Affiliations:** 1 Surgical Outcomes Research Centre (SOuRCe), Royal Prince Alfred Hospital, Building 89, Level 9, Missenden Road, Camperdown, Sydney, NSW 2050 Australia; 2 Sydney Medical School, The University of Sydney, Sydney, NSW Australia; 3 Institute of Academic Surgery (IAS), Royal Prince Alfred Hospital, Sydney, NSW Australia; 4 Faculty of Health Sciences, Discipline of Physiotherapy, The University of Sydney, Sydney, NSW Australia; 5 Department of Physiotherapy, Royal Prince Alfred Hospital, Sydney, NSW Australia; 6 Department of Anaesthetics, Royal Prince Alfred Hospital, Sydney, NSW Australia

**Keywords:** Feasibility, Acceptability, RCT, Pre-operative, Exercise, Surgery, Cancer, Post-operative outcomes

## Abstract

**Background:**

There is a need for evidence of the effectiveness of pre-operative exercise for patients undergoing major cancer surgery; however, recruitment to such trials can be challenging. The PrE-operative Physical Activity (PEPA) Trial will establish the feasibility and acceptability of a pre-operative exercise programme aimed to improve patient outcomes after cytoreductive surgery and pelvic exenteration. The secondary aim is to obtain pilot data on the likely difference in key outcomes (post-operative complications, length of hospital stay, post-operative functional capacity and quality of life) to inform the sample size calculation for the substantive randomised clinical trial.

**Methods/design:**

Twenty patients undergoing cytoreductive surgery and pelvic exenteration at the Royal Prince Alfred Hospital, Sydney will be recruited and randomly allocated (1:1 ratio) to either 2 to 6 weeks’ pre-operative exercise programme (intervention group) or usual care (control group). Those randomised to the intervention group will receive up to six individualised, 1-h physiotherapy sessions (including aerobic and endurance exercises, respiratory muscle exercises, stretching and flexibility exercises), home exercises (instruction and recommendations on how to progress the exercises at home) and encouragement to be more active by using an activity tracker to measure the number of steps walked daily. Patients allocated to the control group will not receive any specific advice about exercise training. Feasibility will be assessed with consent rates to the study, and for the intervention group, retention and adherence rates to the exercise programme. Acceptability of the exercise programme will be assessed with a semi-structured questionnaire. The following measures of the effectiveness of the intervention will be collected at baseline (2 to 6 weeks pre-operative), a week before surgery, during hospital stay and pre hospital discharge: post-operative complication rates (Clavien-Dindo), post-operative functional capacity (Six-minute Walk Test) and quality of life (SF-36v2®) and length of hospital stay. Functional status will be additionally measured using Cardiopulmonary Exercise Testing (CPET), at baseline and within a week before surgery.

**Discussion:**

The PEPA Trial will provide important information about the feasibility and acceptability of a pre-operative exercise programme for patients undergoing major cancer surgery. Data from the PEPA Trial will be used to inform the design, methodology and to calculate sample size required for a larger, definitive trial.

**Trial registration:**

Australian New Zealand Clinical Trials Registry, ID: ACTRN12617001129370. Registered on 1 August 2017.

**Electronic supplementary material:**

The online version of this article (10.1186/s13063-018-2481-2) contains supplementary material, which is available to authorized users.

## Background

For people with major advanced primary or recurrent gastrointestinal malignancies, surgery to achieve complete removal of the tumour, with or without neo-adjuvant or adjuvant chemotherapy, offers the best chance of cure [1, 2]. Major, advanced primary or recurrent gastrointestinal malignancies collectively are the most lethal cancers worldwide. They are also some of the leading causes of disease burden in Australia and some of the most expensive cancers to treat [3]. The burden of these major advanced primary or recurrent malignancies is explained by the complexity and extension of these radical procedures; with some having a treatment cost of around AU$137,000; most of this cost is associated with the extended length of stay in hospital due to the high number of complications [4, 5].

One intervention that holds potential to reduce post-operative complications is a pre-operative exercise programme to improve patients’ fitness before surgery [6, 7]. A recent systematic review of pre-operative exercise interventions for patients undergoing cancer surgery [8] found 17 reports of 13 different trials investigating the effectiveness of exercise in six oncological procedures: colon (one trial, *n* = 42) [9], colorectal liver metastasis (one trial, *n* = 38) [10, 11], oesophageal (two trials, *n* = 98) [12–14], lung (six trials, *n* = 434) [15–21], oral (one trial, *n* = 60) [22] and prostate cancer (two trials, *n* = 134) [23, 24]. While for most cancer procedures only reports of small single trials were found, pre-operative exercise in patients undergoing surgery for lung cancer was shown to reduce the risk of post-operative complications by 48%, and reduced the length of hospital stay by 3 days when compared to no treatment control or minimal intervention. Interestingly, no information on cost and quality of life outcomes were reported.

In other patient groups, promoting physical activity in the pre-operative period appears promising to improve functional capacity and facilitate post-operative recovery [25]. Given that the surgical work-up period for most patients who have major advanced primary or recurrent gastrointestinal cancer is approximately 4 to 8 weeks, there is opportunity to incorporate an exercise programme into the pre-operative preparation period without the need to delay surgery.

Therefore, the *primary aims* of this pilot randomised controlled trial are to determine: (1) the feasibility of incorporating a standardised, intensive exercise programme into the pre-operative period for patients undergoing cytoreductive surgery or pelvic exenteration at Royal Prince Alfred Hospital (RPAH), Sydney; (2) the acceptability of the exercise programme to patients and (3) the acceptability to patients of being randomised to the exercise programme or usual care. The *secondary aim* is to obtain pilot data on the likely difference in key outcomes (post-operative complications, length of hospital stay, post-operative functional capacity and quality of life) to inform the sample size calculation for the substantive randomised clinical trial.

Our hypothesis is that the pre-operative exercise programme in combination with usual care is feasible and acceptable for patients undergoing cytoreductive surgery and pelvic exenteration.

## Methods/design

**Fig. 1:**
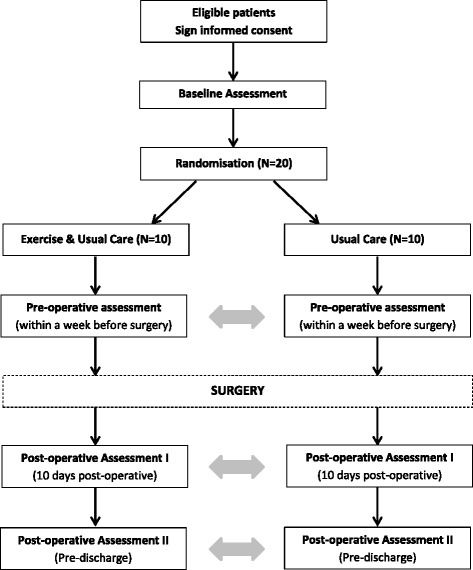
PEPA Trial design

**Fig. 2:**
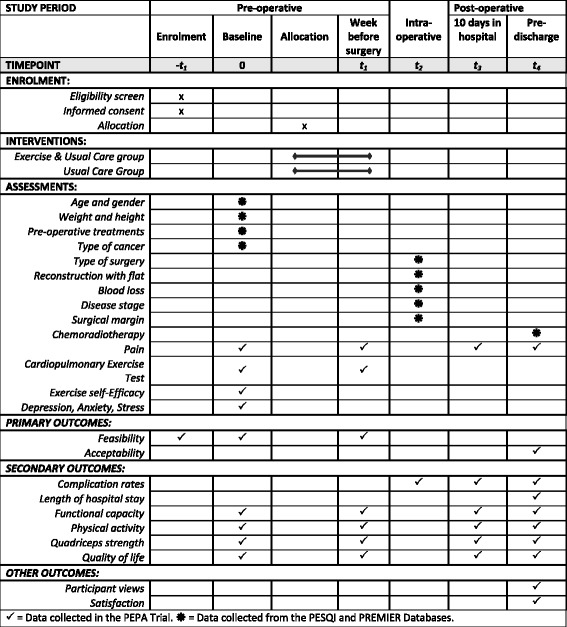
Details of the schedule of enrolment, interventions and assessments according to the Standard Protocol Items: Recommendations for Intervention Trials (SPIRIT) Diagram

The PrE-operative Physical Activity (PEPA) Trial is a two-arm, pilot randomised controlled trial. The design of the trial is shown in Fig. 1. This protocol has been prepared according to the Standard Protocol Items: Recommendations for Interventional Trials (SPIRIT) Statement [26, 27] and will be reported according to the Consolidated Standards of Reporting Trials (CONSORT) Statement [28], and is registered at ANZCTR (ACTRN12617001129370). The SPIRIT Checklist is provided in Additional file 1 and the SPIRIT Diagram is included in Fig. 2.

A total of 20 patients will be randomly assigned via concealed allocation to receive: (1) a pre-operative exercise programme and usual care (intervention group) or (2) usual care alone (control group). To reduce participant burden, this study will use clinical data collected by the Pelvic Exenteration Surgery Quality and Improvement (PESQI) (Protocol No X13–0283 and HERC/13/RPAH/371) and the Peritonectomy Surgical Research Programme (PREMIER) (Protocol No X16–0482 and HREC/16/RPAH/691). Ethical approval for the PEPA Trial was obtained by the Sydney Local Health District Human Research Ethics Committee (Protocol No X17–0189 and HREC/17/RPAH282).

### Setting and participants

All consecutive patients undergoing cytoreductive surgery or pelvic exenteration at the RPAH and attending consultation rooms 2 to 6 weeks prior to their operation will be invited to participate by their treating surgeon.

### Eligibility criteria

The inclusion and exclusion criteria are listed below:

Inclusion criteria: to be eligible for the study, patients will have to be adults aged 18–80 years:

- Undergoing elective cytoreductive surgery or pelvic exenteration
- Present to the participating gastrointestinal surgeon at least 2 weeks prior to planned surgery

Exclusion criteria: patients will be excluded from the study if they have:

- Cognitive impairment such that they are unable to provide informed consent
- Co-morbidity preventing participation in exercise (i.e. major cardiac, respiratory or musculoskeletal disease)
- Inadequate English language to the complete outcome measures
- Current participation in an active exercise programme similar to the proposed intervention
- Unable to attend the exercise programme (e.g. living in another state)

Eligible patients will be provided with the Participant Information Sheet and the Participant Consent Form from their participating surgeon, who will explain the study aims and the practicalities of the exercise programme and data collection. Once informed consent has been obtained, the participating surgeon will forward the signed Participant Consent Form with the patients’ contact details to the study’s research officer who will contact the patient to organise a meeting for baseline assessment and randomisation. Patients will be reassured that their participation in the study will not alter their treatment pathway and that they can withdraw from the study at any time.

### Randomisation and allocation concealment

A research officer not involved in the trial will prepare the group allocation after written informed consent and baseline assessment are obtained. Randomisation will be carried out on a 1:1 basis and will utilise a computer-based, random-sequence generator, stratified by surgical procedure (cytoreductive surgery or pelvic exenteration). Consecutively numbered, sealed, opaque envelopes containing group allocation will be prepared by a researcher officer not involved in the trial. The concealed envelopes will be stored in a locked cabinet and will be opened in sequence to reveal group allocation by a research officer not involved in the recruitment process, treatment, or assessment of outcomes.

## Treatments

### Intervention group: pre-operative exercise and usual care

The exercise programme will consist of 60-min individualised (one-to-one) training session with a registered physiotherapist, once a week, for 2 to 6 weeks (maximum of six sessions). The exercise programme will be tailored to each patient based on a health assessment, taking into consideration patients’ current health status, physical activity level, presence of co-morbidities and medical history. Each session will consist of 10 min of warm-up exercises, 40 min of aerobic and endurance, respiratory and muscle-strength exercises, and 10 min of cool-down activities. The aerobic and endurance exercises will be performed at 12 to 14 on the rated perceived exertion scale (Borg Scale) [29] and the strength training will be performed at an intensity of 40% to 60% of the 1-repetition maximum (Table 1). Participants will be given instructions and recommendations on how to progress the exercises at home (four sessions of 60 min each per week at home). In addition to home exercises, patients will be encouraged to be more active by using an activity tracker (Fitbit™) that will be used to provide real-time feedback about their daily physical activity goals. Patients will be asked to report on a daily basis the number of steps per day and number of home exercises performed during the entire pre-operative period. Home exercises and number of steps will be captured in a daily exercise diary that participants will complete until the end of the exercise programme.

**Table 1:**
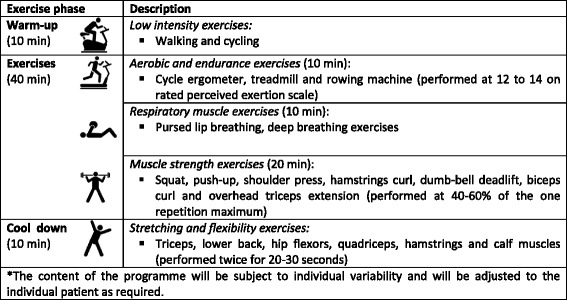
Supervised pre-operative exercise programme (2 to 6 weeks)^a^

^a^The content of the programme will be subject to individual variability and will be adjusted to the individual patient as required

### Control group: usual care

The usual-care group will receive the routine care throughout their cancer pathway, from diagnosis to surgical resection and not take part in any formal exercise programme. No specific advice about exercise training will be offered.

### Study measures

Study data will be assessed at baseline (pre-operative), during the week before surgery (1 week pre-operative), intra-operatively, 10 days post-operative and pre hospital discharge (Fig. 2).

Participating surgeons will record clinical information (including intra-operative complications) on a standardised study data collection form. Baseline and follow-up outcome measures will be collected either by a research officer or by the study physiotherapist who will be blinded to group allocation. All pre- and post-operative treatments (e.g. chemoradiotherapy) for participants in both groups will be recorded by a research officer using a standardised form. The intra-operative data is already collected for all RPAH patients by the Pelvic Exenteration Surgery Quality and the Improvement (PESQI) research programme (Protocol No X13–0283 and HERC/13/RPAH/371) and the Peritonectomy Surgical Research Programme (PREMIER) (Protocol No X16–0482 and HREC/16/RPAH/691).

### Outcome measures

The PEPA Trial will collect the following information (Fig. 2):

1. Demographic and clinical characteristics: prior to surgery (baseline), patients’ height and weight (kg), date of birth, gender, address, and type of cancer will be obtained from the PESQI and PREMIER databases
2. Physical assessment: the physical assessment will be performed by the study physiotherapist and will be collected at baseline, within a week before the surgery, 10 days post-operative and pre hospital discharge. The following outcomes will be assessed:

- *International Physical Activity Questionnaire-Short Form (IPAQ-SF)*: self-reported physical activity will be measured using the IPAQ-SF [30]. The IPAQ-SF will be used to calculate the metabolic equivalent (MET) minutes per week spent in walking, in moderate and vigorous activities and the total MET minutes per week. The amount (minutes per day and number of days per week) of walking, moderate activity and vigorous activity recorded by the IPAQ-SF will be multiplied by a weighting (3.3 for walking, 4 for moderate activity and 8 for vigorous activity) to calculate MET minutes per week in each activity, and these values will be summed to produce the total MET minutes per week. Sedentary behaviour will be determined based on time spent sitting per day (minutes). In addition, participants will be categorised as achieving a low, moderate, or high physical activity level
- *Six-minute Walk Test (6MWT)*: the 6MWT will be conducted in accordance with the protocol of the American Thoracic Society. Participants will be instructed to walk as quickly as possible for 6 min up and down a 30-m, straight, indoor corridor. Standardised encouragement will be given each minute during the test. Oxygen saturation, heart rate and the distance walked will be recorded [31]
- *Isometric Quadriceps Strength Assessment*: quadriceps strength will be measured using a hand-held dynamometer [32]. Patients will be assessed in a sitting position with the knees and hips at 90°. Maximal voluntary contraction force (reported in kilograms) will be assessed as the best of three reproducible manoeuvres
- *Five Times Sit to Stand Test*: lower-limb strength and function will be measured using the Five Times Sit to Stand Test [33]. Patients will sit with arms folded across the chest and with their back against a chair. Patients will be instructed to stand up and sit down five times as quickly as possible. Time taken to complete the test will be recorded by the physiotherapist
- *Cardiopulmonary Exercise Test (CPET)*: the CPET will be conducted using a computer-controlled, electromagnetically braked cycle ergometer (Ergoline VIAsprint 200P, Germany) in accordance with the American Thoracic Society/American College of Chest Physicians (ATS/ACCP) statement [34]. maximal oxygen uptake (VO_2_ max; ml/kg/min) and anaerobic threshold (ml/kg/min) will be recorded at baseline and within a week before the surgery

3. Clinical outcomes: clinical outcomes will be collected intra-operatively and post-operatively from PESQI and PREMIER databases

- Complication rates: will be defined as the total number of complications post-operative and will be reported according to the Clavien-Dindo classification of surgical complications [35]
- Length of hospital stay: will be defined as the duration of inpatient hospital stay with the day of surgery considered as day 0

4. Quality of life, the Exercise Self-Efficacy Scale, the Depression, Anxiety, Stress Scale, and Participants views and satisfaction: quality of life will be measured by the Short Form 36 version 2 (SF-36v2®, Australian English) [36] at baseline, within a week before the surgery, 10 days post-operative and at pre hospital discharge. Exercise self-efficacy (measured with the Exercise Self-efficacy Scale) [37] and depression, anxiety, stress (measured with the Depression, Anxiety, Stress Scale) [38] will be assessed at baseline. Participants’ views and satisfaction with the study intervention will be assessed at pre hospital discharge

### Primary outcomes

#### Feasibility

Feasibility of the intervention will be determined by the number of eligible patients recruited, retention and adherence rates to the exercise programme. The retention rate will be defined as the percentage of individuals who completed the PEPA Trial. Adherence will be defined as the percentage of exercise sessions attended by those who were randomised to the intervention group. Adherence to the exercise programme will be recorded using attendance records (recorded by the study physiotherapist)/participant exercise diaries (recorded by the participant).

#### Acceptability

The acceptability of the exercise programme will be assessed by a semi-structured questionnaire administered at pre hospital discharge.

### Secondary outcomes

Secondary outcomes will include complication rates, length of hospital stay, functional capacity (assessed with the 6MWT), physical activity (assessed with the IPAQ-SF), quadriceps strength (assessed with the Isometric Quadriceps Strength Assessment and the Five Times Sit to Stand Test) and quality of life (assessed with the SF36-v2®). Other outcomes, such as adverse events (defined as any undesirable event such as injury, falls, discomfort, or pain), will be collected throughout the trial. Questions related to participants views and satisfaction with the exercise programme will be asked at the end of the study (pre discharge).

### Blinding

Research officers involved in data collection will be blinded to group allocation. Due to the nature of the intervention, it will not be possible to blind participants and the physiotherapist delivering the pre-operative exercise intervention. However, the physiotherapist conducting the physical assessments in both groups and the surgeon will be blinded to participant group allocation and participants will be asked not to disclose group allocation to their physiotherapist assessor and surgeon. In addition, the statistician performing the statistical analyses will be blinded to group allocation.

### Statistical analysis

All data will be stored in a Research Electronic Data Capture (REDCap) database and statistical analyses will be performed using IBM SPSS Statistics version 22 (SPSS Inc., Chicago, IL, USA).

The proportion of patients who consent to the study and the reasons for non-consent will be summarised. Characteristics of patients randomised to each group will be compared. The number and percentage of intervention-group participants who commenced, completed each component and completed the full exercise programme will be calculated. The proportion of patients who developed major complications and the median length of stay will be compared between the intervention and control groups. For the secondary outcomes (functional capacity, physical activity, quadriceps strength and quality of life) change in scores between baseline, 1 week pre-operatively, 10 days in hospital and pre hospital discharge will be compared between the intervention and control groups to inform future sample size calculations. The overall score of the semi-structured questionnaire will reveal the acceptability of the exercise programme.

### Timeline

Based on the number of cytoreduction surgeries and pelvic exenterations conducted at RPAH each year and on our experience with recruitment rate in previous pelvic exenteration studies, it is expected that three participants be recruited per month, to complete recruitment in 7 months (total *n* = 20). The feasibility and acceptability (primary aims of the study) will be completed in 8 months and the final follow-up assessment and manuscript preparation will be completed in the following 3 months.

### Data integrity and privacy

Consenting patients will be allocated to a unique study identification number. The list that matches individual patients with study identification numbers will be kept secured by the study chief investigators in a separate file on a password-protected computer, which will be based on a secure server hosted by the Sydney Local Health District (SLHD) and supported by Information Management and Technology Division (IM&TD). Study data will be entered into a REDCap database with individuals identified by their study identification number (i.e. in re-identifiable format). Only the study coordinators and investigators will have access to these data. All hard copies of the assessment booklets will be kept in a locked filing cabinet in the locked Surgical Outcomes Research Centre (SOuRCe) office at RPAH. This cabinet can only be accessed by the research personal named in this application. These records will be kept for 15 years after conclusion of the study, in accordance with statutory requirements. At the end of the 15 years, all files, electronic and paper, will be destroyed as per SLHD policy. The chief investigator will be the custodian of the study data and will meet with the associate investigators on a monthly basis to monitor data management. The study chief investigator will adhere to a data-cleaning schedule and conduct regular data audits to ensure data integrity. Basic checks will be conducted to identify data entry errors and any errors identified in this process will be corrected. The chief investigator will manage all personal health information in accordance with the Health Records and Information Privacy Act 2002 (HRIP).

### Data and Safety Monitoring Board (DSMB)

A senior lecturer from the Discipline of Physiotherapy of The University of Sydney will be the principal independent member of the Data and Safety Monitoring Board (DSMB) and will monitor adverse events and adherence to the protocol at regular intervals to ensure the safety of participants. The frequency of DSMB meetings and the stopping rules for the study will be defined a priori in a charter, in consultation with the DSMB members and study investigators.

### Research codes of conduct

The trial will conform to the following guidelines and regulations: the Note for Guidance on Good Clinical Practice (GCP), the National Statement on Ethical Conduct in Human Research and the Australian Code for the Responsible Conduct of Research.

## Discussion

This manuscript presents the protocol of a pilot randomised controlled trial evaluating the feasibility and acceptability of a pre-operative exercise programme for individuals undergoing surgery for cytoreductive surgery and pelvic exenteration. This pilot study will provide preliminary data that will be used to inform the design and methodology of a future phase-3 randomised controlled trial investigating the effectiveness of pre-operative exercise programme on patients’ post-operative complication rates, length of hospital stay, function and quality of life outcomes for patients undergoing major cancer surgery. The results of PEPA Trial has the potential to provide robust data to guide clinical decision-making.

A limitation of the pilot study is that patients cannot be blinded to the intervention, which could bias the self-reported outcome measures (secondary aim). However, measures will be taken to minimise bias. The physiotherapist performing the physical assessment and the treating surgeons will be blinded to group allocation. Furthermore, patients will be instructed not to reveal the group to which they were allocated.

## Trial status

The PEPA feasibility and acceptability trial recruited and randomised the first patient on 10 October 2017. The completion (follow-up of all patients) of the PEPA Trial is planned for June 2018. The results are expected to be submitted for publication at the end of 2018.

## Additional file

Additional file 1: SPIRIT 2013 Checklist: recommended items to address in a clinical trial protocol and related documents*. (DOC 121 kb)

## Abbreviations

6MWT: Six-minute Walk Test
ACCP: American College of Chest Physicians
ANZCTR: Australian New Zealand Clinical Trials Registry
ATS: American Thoracic Society
CONSORT: CONsolidated Standards of Reporting Trials
CPET: Cardiopulmonary Exercise Test
DSMB: Data and Safety Monitoring Board
GCP: Good Clinical Practice
HRIP: Health Records and Information Privacy Act
IM&TD: Information Management and Technology Division
IPAQ-SF: International Physical Activity Questionnaire-Short Form
MET: Metabolic equivalent
PEPA: Pre-operative Physical Activity Trial
PESQI: Pelvic Exenteration Surgery Quality and Improvement
PREMIER: Peritonectomy Surgical Research Programme
REDCap: Research Electronic Data Capture
RPAH: Royal Prince Alfred Hospital
SF-36: Short Form 36
SLHD: Sydney Local Health District
SOuRCe: Surgical Outcomes Research Centre
SPIRIT: Standard Protocol Items: Recommendations for Interventional Trials
VO_2_ max: Maximal oxygen uptake
